# Timing over Tuning: Overcoming the Shortcomings of a Line Attractor during a Working Memory Task

**DOI:** 10.1371/journal.pcbi.1003437

**Published:** 2014-01-30

**Authors:** Jonathan D. Drover

**Affiliations:** Brain and Mind Research Institute, Division of Systems Neurology and Neuroscience, Weill Cornell Medical College, New York, New York, United States of America; Université Paris Descartes, Centre National de la Recherche Scientifique, France

## Abstract

How the brain stores information about a sensory stimulus in working memory is not completely known. Clues about the mechanisms responsible for working memory can be gleaned by recording from neurons during the performance of a delayed response task. I focus on the data recorded during such an experiment, a classic tactile discrimination task. I describe how the observed variability in the firing rate during a trial suggests that the type of attractor that is responsible for holding the stimulus information is not a fixed-point type attractor. I propose an alternate mechanism to a line attractor that allows the network to hold the value of an analog stimulus variable for the duration of the delay period, but rather than maintain a constant level of activity, the cells' firing rate varies throughout the delay period. I describe how my proposed mechanism offers a substantial advantage over a line attractor: The tuning requirements of cell to cell connections are greatly eased from that of a line attractor. To accommodate a change in the length of the delay period, I show that the network can be altered by changing a single parameter - the timing of an executive signal that originates outside of the network. To demonstrate the mechanism, as well as the tuning benefits, I use a well known model of propagation in neuronal networks.

## Author Summary

The ability to retain stimulus information after the stimulus has ceased is important for survival. The term “working memory” refers to storage of stimulus information for a short period of time, so that this information can be recalled for a decision making process. A common way to probe for the cellular basis of working memory is recording of neurons during a delayed response task. This study focuses on one of these studies - the now classic experiments of Romo et al.. This experiment demonstrates that the frequency of a tactile vibration is held in memory using a type of encoding where the cellular output depends monotonically on the stimulus variable (frequency). In this paper, I develop a model that accounts for a number of features found in the data. Using the model, I am able to account for a diversity of cellular responses, as well as variability during a trial. This paper builds on previous modeling studies of this data set. The advance is an executive input that controls the behavior of the network, and reduces the burden of tuning compared to previous models.

## Introduction

In order to survive, animals must be able to receive sensory stimuli and hold this information in memory after the stimulus has ceased. The ability to recall sensory information allows the animal to process information and make decisions, such as fight or flight. Certain areas of the brain are known to play a role in the ability to hold sensory information, but precisely how the information is held is not completely known. This type of memory, where the information from a transient stimulus is stored for a short period of time, for use in a task or recall in a decision making process, is referred to as *working memory*.

In order to probe for the neuronal basis of working memory, recordings of cellular activity are made during delayed response tasks. In these tasks, an initial stimulus or cue is given to an animal, and then removed. The relevant cue information is held in memory for the duration of a delay period. At the conclusion of the delay period the animal is asked to demonstrate memory of the stimulus. This is generally done with a motor response (button push, bar grab/release, eye saccade, etc.). In most delay period studies, the cellular responses during the delay period vary widely, both from cell to cell, and even within one cell across trials. Much attention has been given to this variability [1]–[6].

The work in this paper is motivated by the experimental work done in one such study [1], [2]. In these classic experiments, an animal was presented with a tactile stimulus, a vibration briefly applied to a finger. After a delay (3 seconds), the animal is presented with a second stimulus. The animals' task is to correctly signal which of the two frequencies was higher. Thus, for successful completion of the task, the animal is required to hold the frequency of the first vibration (the analog stimulus variable) in memory for the duration of the delay period. Consequently, recordings made during the delay period provide clues about the mechanisms responsible for storing this stimulus variable.

The neuronal correlate of working memory is presumed to be persistent cellular activity [7]–[9]: meaningful neuronal activity that continues after the causal stimulus is removed. Often, the level of persistent activity - the firing rate of the relevant cells - depends on the stimulus itself, and therefore can encode information about the stimulus identity. One example, and a focus of this paper, is the case of monotonic encoding. This type of encoding refers to a scenario in which the level of cellular activity depends monotonically on an analog stimulus variable, such as the frequency of a tactile vibration.

The recordings [1], [2] show that there are cells in the frontal cortex that have a monotonic relationship with the stimulus frequency. A structure commonly used to model this type of relationship with a continuous variable is the line attractor [10]. However, perfect line attractors are unlikely to exist in nature as they require exact tuning. Moreover, even if a perfectly tuned line attractor was possible, they cannot stably hold information since they are only neutrally stable along the axis of the attractor, allowing for corruption by noise [11].

A number of features found in the data further suggest that a true line attractor is not the correct type of attractor. There is a lot of variability in the data, both from cell to cell, and at the single cell level. The first type of variability is a large diversity of behaviors among cells. The authors [1] divide cells into three classes - early, persistent, and late. The classification refers to when, during the delay period, the cell is monotonically tuned to the stimulus variable. Early cells are those cells that encode the stimulus during the first part of the delay period, but then lose tuning with the stimulus. Late cells do not begin the delay period tuned to the stimulus, but they are activated and are monotonically tuned to the stimulus at the end of the delay period. The persistent cells are monotonically tuned to the stimulus variable for the entire delay period.

This division in behavior results in a second feature of the data - a systematic change in the number of cells that encode the stimulus at any given time. At the start of the delay period, both early cells and persistent cells are tuned to the stimulus. As the early cells fall out of tune the number of encoding cells decreases, until only persistent cells represent the stimulus variable. As the late cells become tuned to the stimulus, and the persistent cells remain tuned, the total number of encoding cells grows. These changes generate the U-shaped curve describing the number of cells encoding the stimulus as a function of time [1].

The third important feature of the data is the variability of the firing rates during the delay period. It is evident from the experiments that the persistence is not a fixed-point type of persistence, where the cell assumes an invariant firing rate. In these persistent cells, the level of persistent activity is not generally constant for the duration of the delay period. Rather, persistent cells demonstrate changes in their activity level during the course of the delay period. Still, these cells maintain a monotonic relationship with the stimulus variable.

I will show how these observations suggest two things: First, the changes in the number of encoding cells, and the division into early, persistent and late, suggest that the neural representation of the stimulus variable is held as a wave. Second, the variations during the delay period are the result of a poorly tuned line attractor combined with a time-aware correction mechanism to account for the imperfections.

I aim to describe how a network of cells that are not tuned well enough to act as a line attractor can still hold a signal for the duration of a delay period. The key ingredient is a time aware task input [12] that allows the network to amplify its activity, correcting for the deviation of the tuning from that of a line attractor. I also show how this network of cells can be tuned to delay periods of different length by changing a single parameter - the timing of the task input - rather than by manipulating the cell-to-cell connectivity. This task input is assumed to be an executive input originating from outside of the network.

## Models

My goal is to describe a mechanism that can account for the cellular activity recorded during the tactile discrimination task. The key aspects of the mechanism are traveling wavefronts. In choosing an illustrative model, I only require that the model admit traveling wave solutions. I use a biologically motivated model of propagation between cells [9], [13]. This model admits traveling wave solutions, but is otherwise generic:
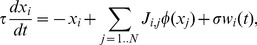
(1)where 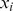 is the activity variable, 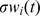 is the noise for the 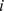
th node (modeled as a Wiener process), and 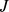 is the matrix of connection strengths (The element 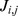 is the connection strength from node 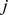 to node 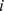). The function 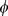 is a response function, converting presynaptic activity to postsynaptic input. This function is very important for the mechanism that I propose, and is discussed in detail below (section 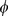 and the task manipulations).

I assume feed forward, nearest neighbor connectivity, ie.
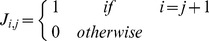
(2)This type of connectivity is chosen for its simplicity, as well as the existence of traveling wave fronts. The results that we obtain here generalize to more complicated types of connectivity. Later, I will demonstrate the mechanism for a model where the connectivity matrix, 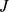, is completely symmetrical.

### 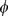 and the task manipulations

The function 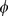 is central to the mechanism. Manipulation of this function is how external events (stimulus information, for example) influence the network. In this section, the different roles this function fills are outlined.

**Figure 1:**
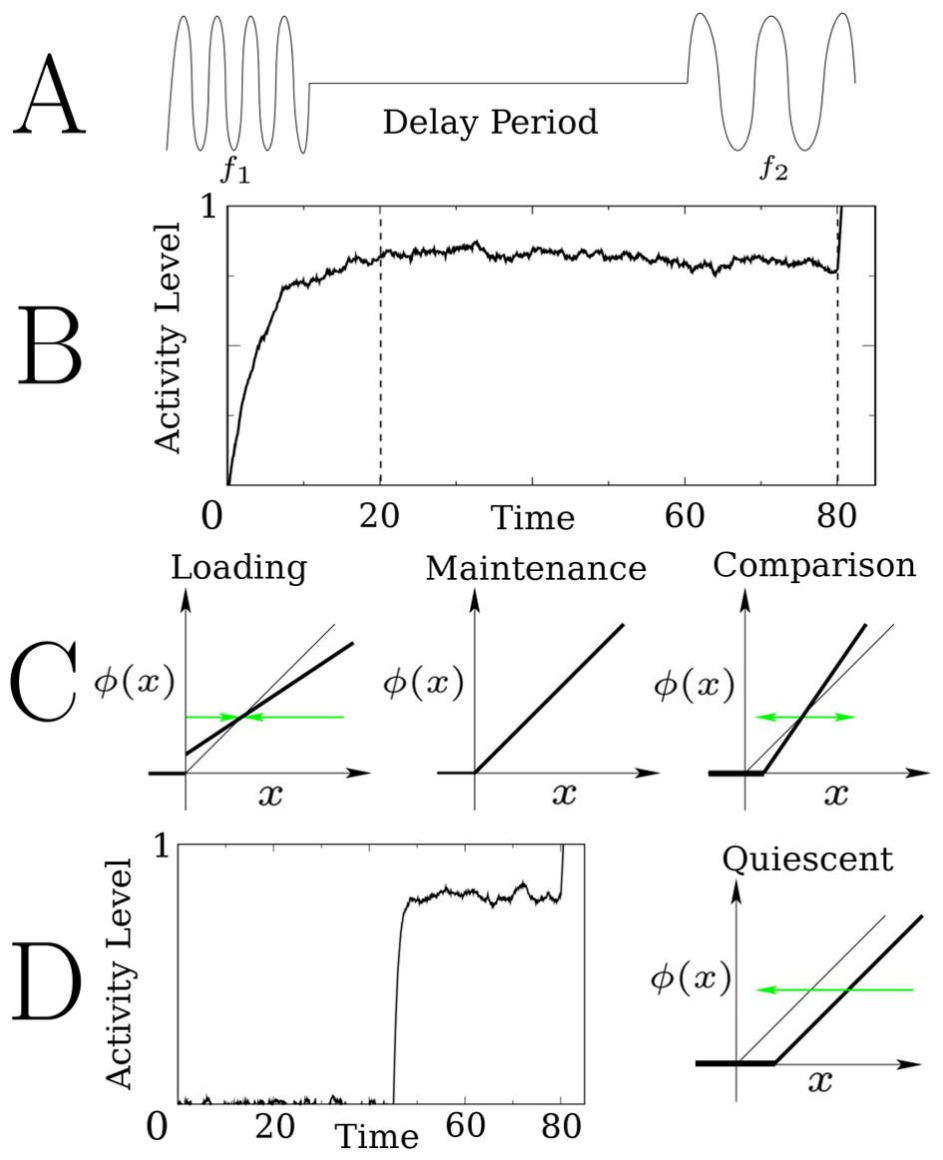
Components of the proposed memory mechanism. A. The delay tactile discrimination task. B. The time series of a persistent cell's activity (equations (1–2)) during the three components of the task, as described by Machens et al. [7]: Loading, maintenance, and comparison. Dotted lines delineate the times (stimulus off at 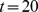 and the comparison is made at 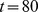) when the network changes between these components. C. Diagrams showing the gain function 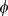 (bold curves) for the loading, maintenance and comparison components of the task. Green arrows show the direction that the activity will move, relative to the fixed point, during the respective component of the task. Note that 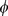 is never negative, and defined as zero when 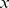 is less than threshold. D. Shown is the time series of a cell that is held quiescent, a component of the task where input to a cell is ignored. Upon receipt of an external input, the cell switches to the maintenance configuration, and assumes the activity level of the persistent cells. This activity level is maintained until the end of the delay period, when the network enters the comparison phase of the task.

There are four different configurations of 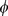. Three of these parallel the “loading”, “maintenance”, and “comparison” task components described by Machens et al. [7]. The fourth role of the 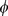 function is to prevent a cell from responding to input (“quiescent”). Figure 1 shows the four different configurations of 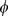: a stable equilibrium point (Row B, left), a line attractor (Row B, center), an unstable equilibrium point (Row B, right), and a quiescent mode (Row D, right). Each of these configurations corresponds to a subtask of the working memory task. The first three are implemented as Machens does [7]: The stable fixed point is for loading the stimulus variable into the network. The maintenance configuration is a line attractor. The comparison configuration is an unstable fixed point.

Chronologically, the first component of the task is “loading” the stimulus variable into the network. During this component of the task, the network is exposed to a stimulus that is described by an analog scalar variable. Here, this variable is the frequency of the tactile vibration. Since the cells tune monotonically to the stimulus variable, the cellular response to the stimulus will be a monotonic function of the stimulus variable. In terms of 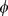, this is done by creating a stable fixed point at the desired activity level (left panel of item C in figure 1). The activity level at this fixed point is determined by the slope of 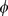. Thus, the slope of 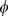 is a monotonic function of input frequency. So, for a cell that is positively monotonically encoding the stimulus, the slope of 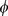 is a monotonically increasing function of frequency. The stable fixed point draws the activity toward this frequency specific level, and the stimulus variable is loaded into the network.

The second component of the task is “maintenance”. This begins once the stimulus is removed. This is the actual memory component of the task, where the network contains information about a stimulus that is no longer present. The information is to be retained for the duration of the delay period. In terms of 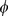, this configuration is shown in the center panel of item C in figure 1. In this configuration, the network behaves as a line attractor. The line attractor holds the stimulus dependent values throughout the delay period. The important result of this study is how the brain might overcome the drift that occurs when this configuration is not perfect - when the lines do not perfectly overlap.

The final component of the task is “comparison”. This component occurs at the end of the delay period. In the experiments, a second stimulus of frequency 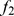 arrives and the objective for the animal is to compare this stimulus to the original stimulus (frequency 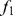). In terms of 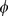, this configuration is shown in the rightmost panel of item C in figure 1. There is an 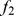-dependent unstable fixed point, where the slope of 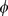 is determined by the stimulus frequency. This unstable fixed point acts as a separatix. If the 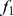-dependent activity levels are above this separatix, they will increase. Conversely, if the 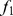-dependent levels are below this separatix, they will quickly decrease. So, whether the activity level increases or decreases upon the arrival of the second stimulus determines whether the network assessed the first stimulus frequency (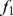) to be higher or lower than the second (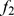), thus providing a comparison.

The right panel of item D in figure 1 shows the “quiescent” configuration. With this 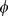, the cells do not respond meaningfully to input. All activity will quickly decrease to zero. The usefulness of this configuration is described in the next section.

## Results

Earlier, I described three characteristics of the data recorded during a delay response task [1], [2]. These features suggest a mechanism that the brain can employ to store an analog stimulus variable for the duration of a delay period. These features are:

1. During the delay period, there is a diversity of behaviors exhibited. There are cells that are monotonically tuned to the stimulus only during the beginning of the delay period (early cells), during the entire delay period (persistent), and only during the end of the delay period (late cells).
2. The number of cells monotonically tuned to the stimulus decreases during (roughly) the first half of the delay period. Near the midpoint of the delay period, this number begins to increase, and continues to increase until the end of the delay period.
3. The firing rate of persistent cells during the delay period is not constant. At the start of the delay period, the firing rate of the persistent cells is tuned to the stimulus variable. This activity level does not remain constant, however. During the delay period, the firing rate varies. Though they do not maintain a constant firing rate, persistent cells maintain a monotonic relationship with the stimulus variable for the duration of the delay period.

In this section, I show how each of these features shape the proposed memory mechanism. I divide the section into three parts, as itemized above. Though done sequentially, it will become apparent that, in my interpretation, these features are tightly intertwined. Once the model is built, I will show how this model is tuned, and why this provides a substantial advantage in feasibility, with regard to tuning, over a regular line attractor.

The keystone of the mechanism is an input that originates externally to the population of cells that we focus on. This input is an executive one, and provides an interpretation of time to the network. The assumption that the timing originates externally is supported by the data. Machens et al. [14] use principal component analysis to show that the cellular responses to the stimulus can be divided into two groups - those components whose variance is due to the stimulus frequency and those components whose variance is due to time. The authors show that the variance due to time is external to the network. The timing of this task input determines the behavior of the network and can be used to tune the network without changing any of the intrinsic properties of the network (eg. connection strength between cells).

### Early, persistent, and late cells

**Figure 2:**
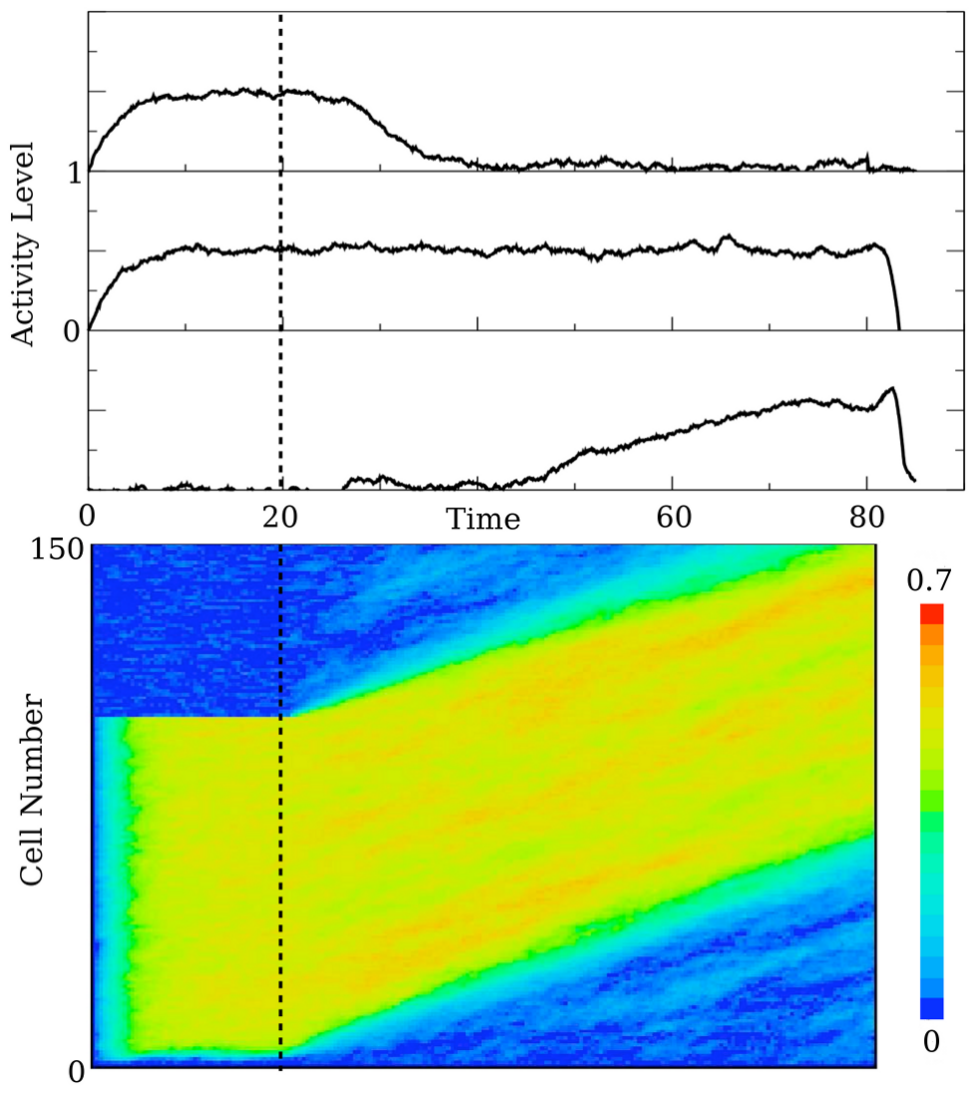
A traveling pulse as a mechanism for early, persistent and late type cells. The top panel shows three time series from different locations on the chain. The top graph is the time series for an early cell, or a cell that ceases to encode shortly after the delay period has begun. Below it is a persistent cell, a cell that maintains a relationship with the stimulus throughout the delay period. Below this is the time series for a late cell. The array plot (bottom) shows the pulse in space (cell number, vertically) and time (horizontal). For all panels in this figure, 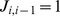, so that the network behaves as a line attractor. To generate the wave, the first 100 cells (out of 150) are stimulated. The noise parameter is 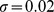.

I begin by demonstrating how a traveling wave can account for the division of the population into early, persistent, and late cells. Figure 2 shows a solution to equations (1) (2). The array plot shows the solution as a function of time and position in the chain. Also shown are temporal profiles of three cells - one that loses activation quickly (early), one that is active throughout the delay period (persistent), and a cell that does not tune to the stimulus variable until the end of the delay period (late).

Figure 2 demonstrates how a traveling pulse can generate a pattern that would allow cells to be classified as early, persistent, or late. There are actually a pair of wavefronts - a leading wave front and a trailing wavefront. The leading wavefront tunes cells to the stimulus variable. Cells lose their relationship with the stimulus as the trailing wavefront passes. By definition, the arrival of the stimulus tunes the early and persistent cells to the stimulus variable. What separates early cells from persistent cells is the position in the chain. Early cells are first in the chain, and the trailing wavefront passes through these cells early in the delay period. Persistent cells are further along in the chain, and the trailing front does not reach these cells during the delay period. Late cells, in this illustrative scenario, are later in the chain than the persistent cells. They are not tuned to the stimulus initially, rather they only become tuned as the leading wavefront reaches them.

Of importance is that the leading and trailing wavefronts have the same slope in the array plot of figure 2. Thus, these wavefronts have the same speed causing the number of cells encoding the stimulus to be constant throughout the delay period. This is not what the experiments show. Rather, the number of cells encoding the stimulus variable decreases at first and then, at some point near the middle of the delay period, begins to increase. The simple pulse described so far is not capable of this. In the next section, I discuss a modulation of the leading wavefront that can account for the initial decrease in the number of encoding cells.

### Decreasing and increasing number of encoding cells

The second feature of the data that was identified was a systematic decrease in the number of encoding cells during the first half of the delay period, followed by an increase. In the previous section, I showed how a pair of traveling wavefronts can account for the existence of early, persistent, and late cells. The problem remaining at the end of the section was that the trailing and advancing wavefronts have the same speed, and so the number of encoding cells is constant. In this section I describe how the wavefronts can be modulated during the delay period to account for this characteristic.

By definition, the initial decrease in the number of encoding cells is due to the early cells losing their monotonic relationship with the stimulus. Similarly, the subsequent increase can only be due to the late cells assuming a stimulus dependent activity level. Thus, the transition from decreasing to increasing number of encoding cells is equivalent to the transition from early cell decay to late cell activation. An important result from the experiments [1], [2] is that the transition from decreasing to increasing occurs roughly halfway through the delay period. This is regardless of the length of the delay period. To illustrate, the authors show what happens when the length of the delay period is changed from 3 seconds to 6 seconds. They show that the late cell response, which began roughly halfway through the delay period for the 3 second delay period, is stretched to roughly halfway through the 6 second delay period after a couple of trials. So, a change in the length of the delay period modulates the time that the transition from decreasing number of cells to an increasing number of encoding cells occurs.

I suggest that the external executive input is responsible for the transition in the number of encoding cells. Suppose that, prior to the arrival of this input, the late cells are not allowed to tune the stimulus variable. The effect of the executive input, then, is to allow the late cells to participate in the task. Prior to the arrival of the input, the leading wavefront is frozen. The early cells are simultaneously falling out of tune, and so the net result is a decreasing number of encoding cells, prior to the arrival of the input.

To incorporate these changes into the model (1), I need to specify 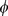 for the late cells, separately from the early and persistent cells. I do this by designating the late cells as “quiescent”, (Figure 1), prior to the arrival of the executive input. When the input arrives, its action is to shift the configuration from “quiescent” to the “maintenance” configuration (Figure 1). This switch allows the leading wavefront to advance into the late cells, tuning them to the stimulus variable.

**Figure 3:**
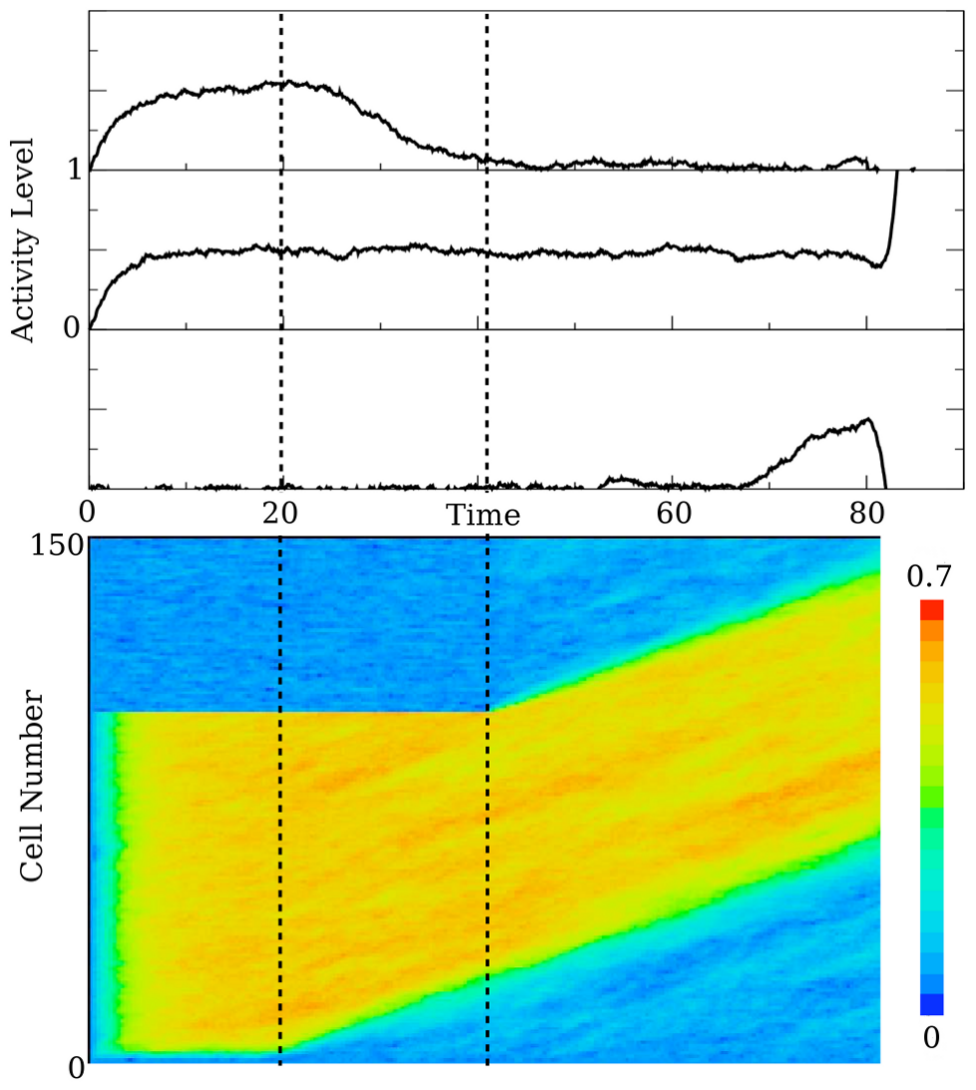
The modified wave. The panels describe the same things as in figure 2, with the same parameter values. The removal of the stimulus is given by the first dotted line. The arrival time of the task input is represented by the second dotted line. Of note in this figure is that the late cells are held back, and do not participate in the task until the task input arrives.

Figure 3 shows the network with the late cells modulated as above. Notice the frozen leading wavefront for the first half of the trial. Prior to the inclusion of the late cells, The number of encoding cells decreases. When the activity is allowed to propagate into the late cells, they become tuned to the stimulus. A difficulty also arises: the persistent cells are losing their monotonic relationship with the stimulus and so there is no net gain in the number of encoding cells. The experiments clearly show an increase in the number of encoding cells. To account for this, either the advancing front (the front going into the late cells) must be faster that the trailing front, or the trailing front (the front that causes the early cells to fall out of tune) must slow down.

Simply increasing the speed of propagation in the late population, thus speeding up the advancing front, would accomplish the growth in the number of encoding cells, but the data suggests that this is not the case. Persistent cells are also impacted by the arrival of the executive input. Many persistent cells show a dramatic change in behavior simultaneously with the incorporation of the late cells. It is then natural to suspect that the executive input is involved with this change of behavior. I posit that the late cells project back onto the persistent cells, freezing the trailing wavefront, and allowing the number of cells that encode the stimulus to increase. The change in the behavior of persistent cells is the topic of the next section.

### Varying firing rates for persistent cells

Persistence, as a mechanism, is a staple of working memory. It is how cells can hold information about the past - a stimulus that is no longer present. Persistence is often modeled as a fixed point, or for the case studied here - monotonic encoding - a line attractor. Each of these attractors holds a cellular variable (eg. firing rate) constant for the duration of the delay period. However, the persistent cells recorded in many working memory studies do not behave as a fixed point. Rather, the firing rates of persistent cells vary widely during the delay period. For the experiments that are the focus of this paper, the large variation of the persistent cells is divided into four categories - cells that initially decrease and then increase, cells that decrease for the duration of the delay period, and the opposite behaviors. Here, I only consider those persistent cells that initially decrease, and after the executive input, are amplified (a typical example is shown in Figure 2 of [1]).

A line attractor is often used to store an analog variable [7], [10], but this mechanism requires very precise tuning. However, a network that admits a near-line attractor (a line attractor where the tuning is not perfect) is still capable of maintaining a monotonic relationship with the stimulus variable. That is, for two stimuli 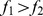, and firing rates 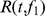, 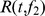, it may be possible to tune the network well enough so that 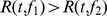 for all 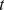 in the delay period, even if 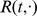 is not constant. This is possible if the tuning is close to that of a line attractor, but not perfect. The imperfections will result in a slow drift from the original value, as shown in [10]. If the tuning is good enough to make this drift sufficiently slow, then a monotonic relationship between the stimulus variable and the cellular output over the course of the delay period can be achieved.

In the model, the cell-to-cell connections are determined by the entries of the connectivity matrix, 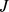 (equation (1)). We are assuming nearest neighbor, feed forward connectivity, so all non-zero entries reside on the first sub-diagonal (equation (2)). For a perfectly tuned line attractor, each of these entries are 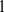. The slow drift is modeled by allowing these entries to be less than 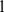 (the slow drift will be a decreasing one).

The change in connection strengths will cause the amplitude of the wave to decrease slowly toward zero. If the entries of 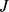 are close enough to 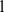, the exponential decay will be slow and monotonicity will be preserved. (**Note:** If the initial stimuli are very close in scalar value, and there is sufficient noise, this monotonicity can be broken. For example, if in one trial the stimulus is at 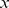 Hz and in another trial the stimulus is 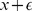 Hz, for a small value of 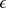, one would expect the noise to destroy the monotonicity. Accordingly, there is a minimum separation between the frequency of the first and second stimuli in the experiments).

In order to reflect the stimulus information, the signal must be amplified to recover from the initial decay. The switch from decreasing activity to increasing activity takes place at nearly the same time the late cells begin to encode the stimulus, so it is natural to view the late cells as implicit in this transition. I propose that the late cells project back onto the early and persistent cells. This feedback accomplishes two things: 1). It amplifies the decayed signal so that the firing rate at the end of the delay period is indicative of that at the beginning - the stimulus induced value - and 2). it stops the trailing wave front from advancing, allowing the total number of cells encoding the stimulus to increase. This addresses the issue that I ended the previous section with - the leading and trailing wavefronts no longer have the same speed.

In order for the feedback from the late cells to amplify the signal, the strength of this feedback must be sufficiently strong. As in [9], the solution of the 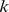
th cell in the chain, after the stimulus has been removed, is given by
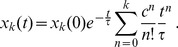
(3)Following Goldman [9], the late cells become part of the wave roughly one time unit for each connection in the chain after the initial blockade (an implicit assumption is that the time constant of a cell is much shorter than the length of the delay period). Once the propagation of activity is allowed to advance into the late cells, they assume this solution as well. So, in general 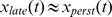 for 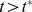, the onset time of the task input. After the inclusion of the late cells, we can approximate the evolution of a persistent cell with
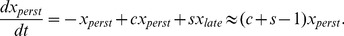
(4)where 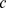 is the strength of input from other persistent cells (the value of 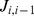) and 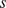 is the strength of the feedback from the late cells. If 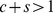, there will be amplification. I will derive the specific restraints on these parameters in the next subsection, and their relationship to the tolerance in the timing of the executive input.

**Figure 4:**
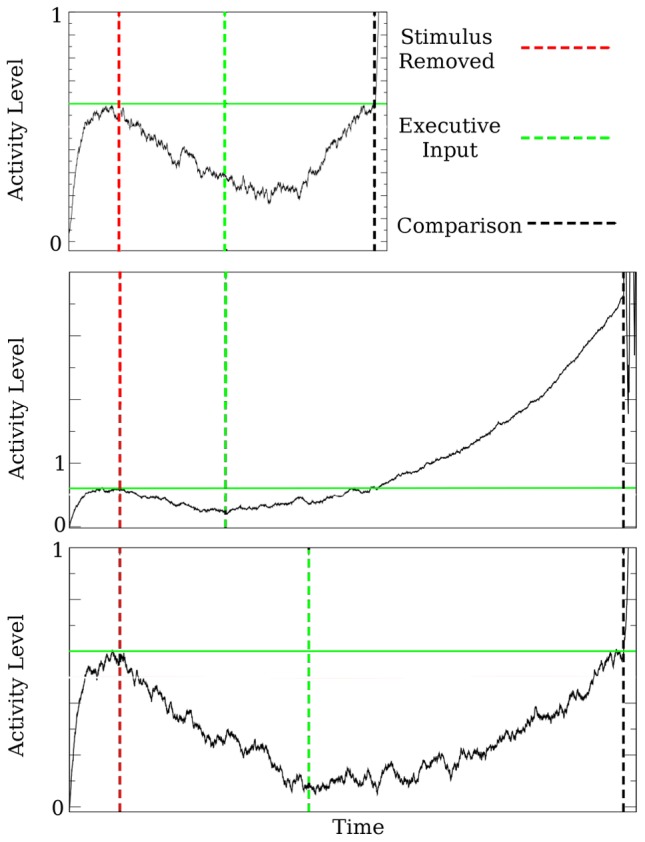
The network is able to tune to different delay period lengths by changing the timing of the task input signal. The top plot shows the evolution of a properly tuned persistent cell during a delay period of length 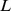. The value of the stimulus variable is 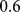 (indicated by the green horizontal line). The second panel shows the same cell, with the same task input timing as the top panel, but with a delay period that is twice the length (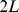). This results in an unreliable representation of the stimulus variable. The bottom panel shows the same cell when the timing of the task input is correctly tuned for the longer delay period. In this figure, 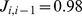, and the feedback strength is 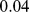. The noise strength is 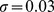.

It is my claim that the decay and amplification mechanisms can greatly ease the cell-to-cell connectivity restrictions of a line attractor. There is another important advantage to the proposed mechanism - it can be used to tune the network to delay periods of different lengths without changing any of the individual connections between cells. Figure 4 shows a demonstration of this. In the top panel of figure 4, the network is tuned so that it successfully stores the stimulus value for a delay period of length 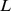. If, on the next trial, the delay period is increased without warning or preparation, say doubled (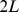), the task input will not move, resulting in an unreliable cellular response. However, after a couple of trials, the timing input is shifted to a time that results in correct trials (bottom panel of figure 4). This is consistent with the data; as reported in [1] there is a slight increase in the error rate directly after the switch from 3 to 6 seconds. After a few trials, the animal's performance improves. Moreover, raster plots show that the onset of late-cell activity gradually adapts to the longer delay period length, after a few trials [1].

This mechanism is further supported by the data. Brody et al. [1] utilize a descriptive model to determine to what extent the activity during the 6 second delay period is a stretched version of the activity during a 3 second delay period. They show that the early cells behave in much the same way for each delay period length. That is, the time course for an early cell does not stretch or contract. The model agrees with this, since an early cell for the 6 second delay period will evolve the same way as it would for a 3 second delay period. In either case, the trailing wavefront has passed. The authors show that the timing of the late cells is stretched by a factor of 2. This also agrees with the model, since the timing of the task input - which determines when the late cells become active - is roughly halfway through the delay period. Doubling the delay period will then roughly double the time at which the late cells begin to encode, yielding an approximate stretch factor of 2.

At the end of the previous section, I concluded that the external signal also causes the freezing of the trailing wavefront, so that persistent cells can remain active. How the trailing wavefront is frozen is interesting, and may not be immediately obvious. The late cells feed back onto all of the early and persistent cells. The early cells are those cells that, by definition, have lost their monotonic relationship with the stimulus. In other words, they have decayed below the level where signal can be differentiated from noise.

**Figure 5:**
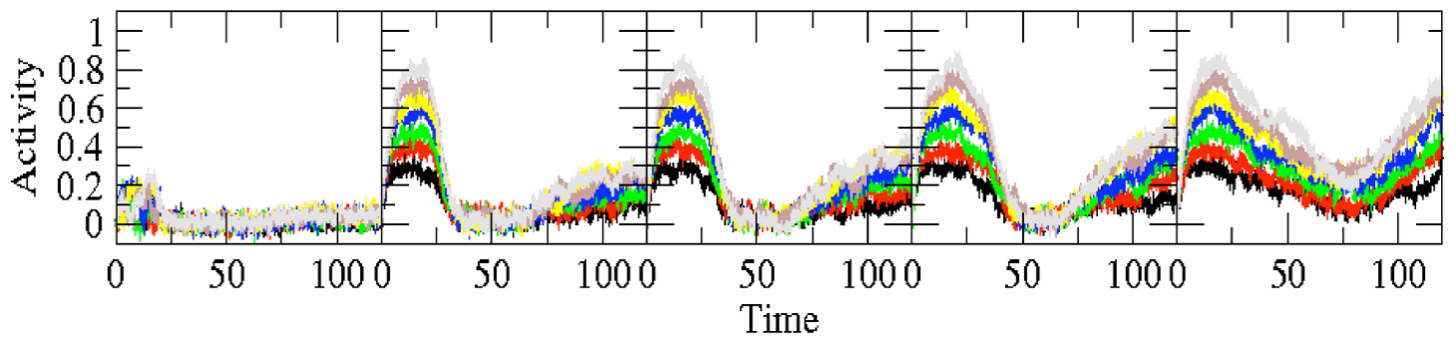
Summation of feedback to freeze the trailing wavefront. Shown are cells 2,12,17,25, and 60. The rightmost plot is the only plot of a persistent cell. All other plots show loss of relationship with the stimulus during the delay period. The summed noise of the early cells, along with a small amount of feedback from the late cells, freezes the trailing wavefront. In this figure, 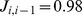, the late cell feedback has strength 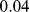, and 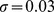 (noise strength).

Still, with the addition of late cell input, the noise in the early cells' activity is amplified and propagates through the medium. Though it is summed noise and does not have a relationship to the stimulus, it does generate enough activity to “prop up” the trailing wavefront. This allows the persistent cells to maintain their monotonicity with the stimulus variable, freezing the trailing wavefront. Figure 5 shows early cells at different locations in the chain, and how they contribute to the maintenance of a persistent cell.

**Figure 6:**
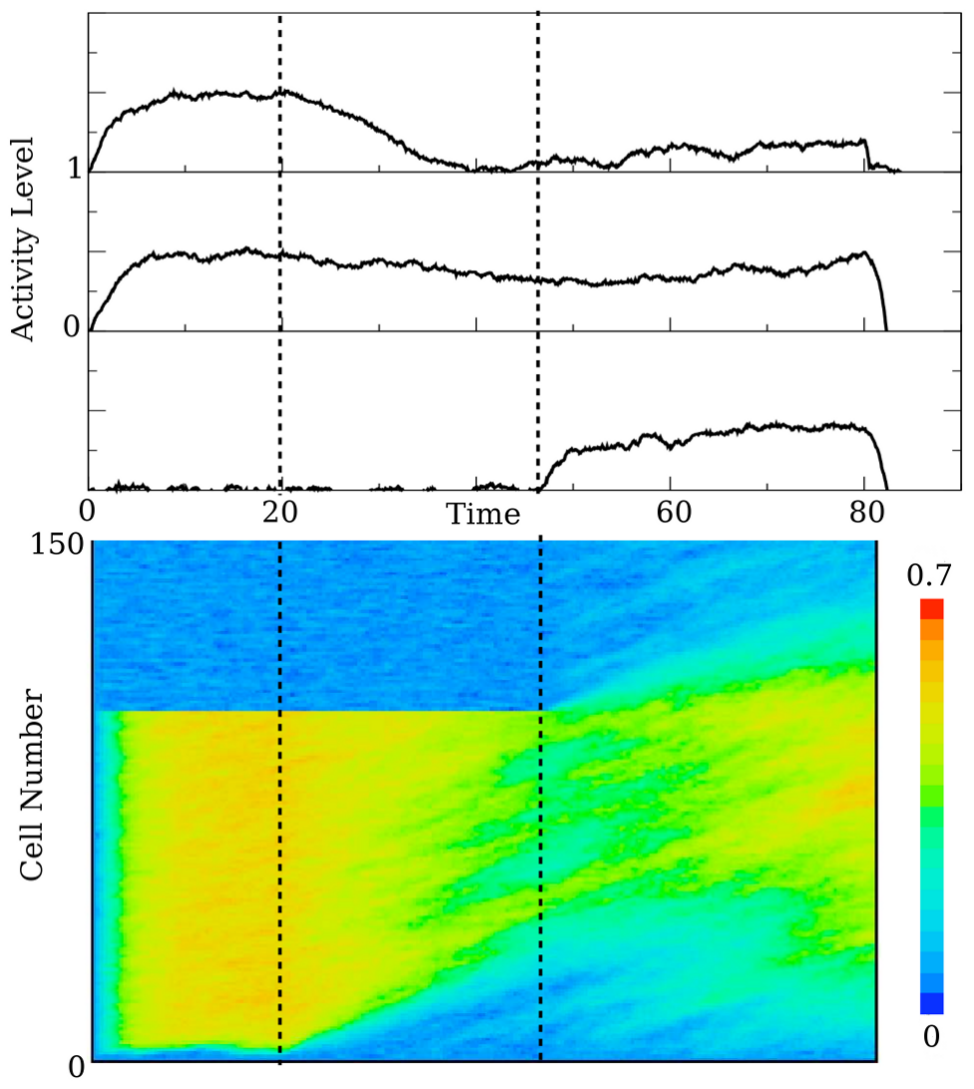
The decay and correct mechanism. The figure is laid out the same as figures 2 and 3. The top panel contains three time series showing the behavior of the three types of cells we consider - an early cell (top), a persistent cell, and a late cell. The panel at the bottom of the figure is an array plot that shows the evolution of the entire network as a function of time. This figure clearly demonstrates the change of behavior for the network after the executive input has arrived - the frozen wavefront prior to the executive input, the activation of the late cells, and the freezing of the trailing wavefront. For all panels in this figure, the feed forward connection strength is 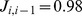 and the feedback from the late cells has strength 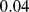. The stimulus turns off at time 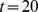, and the executive input occurs at 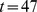. For the noise 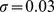.

**Figure 7:**
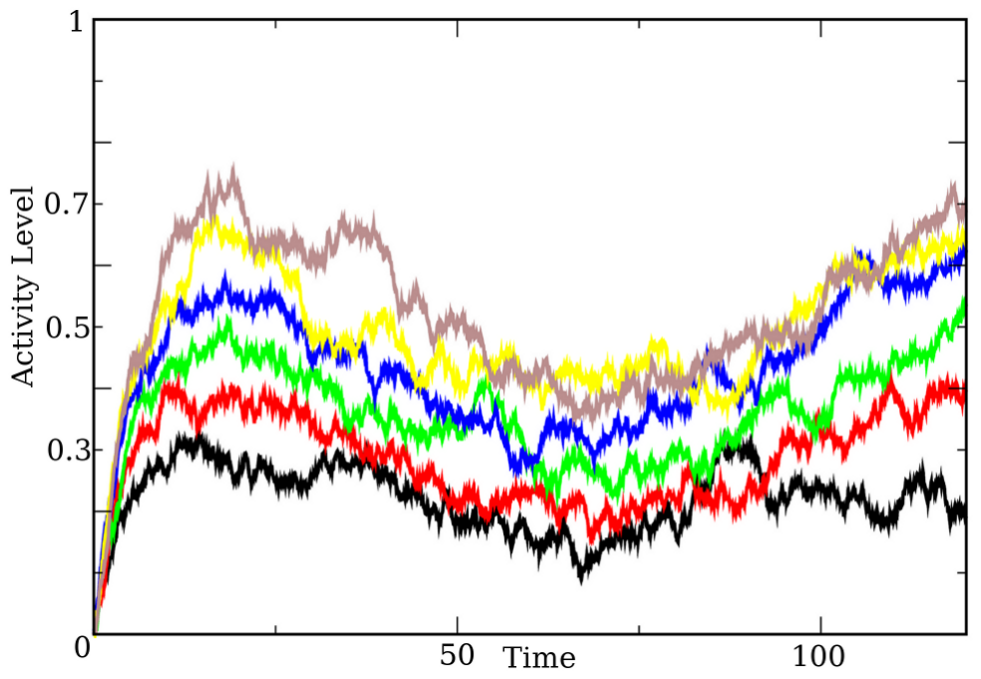
Time series showing the activity level of a persistent cell (used is the last persistent cell in the chain) undergoing the proposed decay-amplify mechanism. Shown are time series for stimulus variable values ranging from 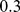 (black) to 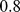 (brown). For each of the simulations, the stimulus was removed at 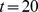, and the executive input arrives at 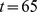. The connection strengths are 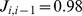 and the feedback is 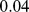. For the noise, 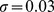.

Figure 6 shows a simulation of the full network. I implement the feedback from the late cells to the early and persistent cells as a connection from a single late cell, though more general patterns would work as well. As the late cells are incorporated into the network, the persistent cells begin to increase their firing rates. The time courses for an early, persistent, and late cell are also shown in figure 6. The decreasing and increasing of the firing rate in a persistent cell is clearly demonstrated, as is the match between the initial stimulus dependent activity level and the level after amplification. Figure 7 shows how this this tuning works for a range of stimuli.

### Comparison to a line attractor

In this section, I show that the proposed mechanism offers a substantial advantage over a true line attractor in terms of the tuning requirements for the connections between cells; i.e. the closeness to the line attractor configuration in figure 1.

The first step is to determine, as a function of the decay and amplification rates, an interval during which the executive input must arrive to correctly amplify the cellular activity. The goal is to determine the length of this interval as a function of the decay and amplification rates (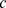 and 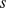 in equation (4)). I show that, for decay (and amplification) rates well outside those acceptable for a true line attractor, this interval of times is within the abilities of networks in the brain.

To quantify the tuning requirements in the context of our model, I first establish how accurate the network needs to be. I define 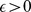 as the resolution of the network. That is, the network has to be able to differentiate between stimulus variable values that are separated by more than 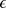. Frequencies closer than this are assumed to be too close to differentiate. Therefore, it is necessary to determine when the task input must arrive so that
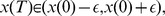
where 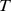 is the length of the delay period. Assume a decay rate of 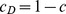, and an amplification rate of 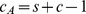, where 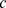 is the coupling strength and 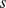 is the strength of the feedback connections, as in equation (4).

First, we derive the requirements on the coupling strengths that are necessary so that a line attractor can hold the value of the stimulus variable for the duration of the delay period. Following Goldman [9], the 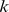-th cell in a chain of cells satisfying equation (1) has the solution
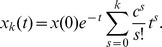
For large 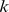, this can be approximated by
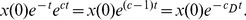
(5)For the regular line attractor, we require that
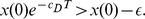
Solving for 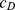 yields
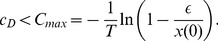
(6)

For the proposed mechanism, the tuning requirement is on the timing of the external task input. To determine this requirement, we determine the allowable values of the task timing (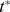) given the rate of decay, and the amplification rate due to feedback from late cells. With decay followed by amplification, equation (5) can be extended to yield
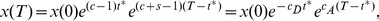
(7)Where 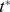 specifies the timing of the executive input. We require 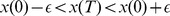. Solving for 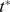 gives
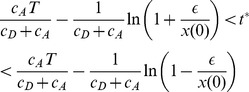
(8)The length of this interval is
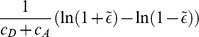
where 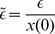. From this inequality, one can see that the length of the interval scales with the sum 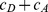. In other words, doubling the sum 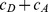 will decrease the length of the interval by a factor of 2.

Now, to show that this is advantageous over tuning a line attractor, we consider how the bound on 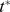 varies as the decay rate increases past the limit allowed by a line attractor. Inserting the bound (6) into the expression for the length of the interval, and letting 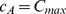 gives
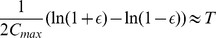
This means that for any choice of 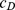 and 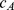, the length of the timing interval will be, approximately,
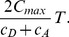
(9)Thus, this mechanism is feasible for values of 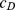 and 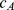 well outside of values that will yield an effective line attractor. As an example, if 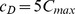 and 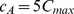, then the interval where the external signal can arrive has length 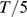. As another example, if 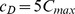 and 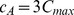 then the interval will have length 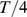. For a 3 second delay period, these examples give interval lengths of 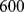 ms and 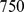 ms, respectively. Based on measured dynamics of neural operations in the brain, these intervals are within the limits of feasibility.

In addition to the length of the interval, equation (8) gives where, during the delay period this interval resides. The interval is centered at
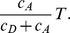
Thus, if 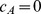 the amplified network acts as a line attractor, and so the timing input should arrive right away. Accordingly, the interval hugs the left endpoint of the delay period. On the other hand, if 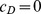, then the network is a line attractor during decay, and the executive input never needs to arrive. Thus, if either the decay or the amplification meet the requirements of a line attractor, then the network is, by default, accurate enough.

### Model performance

In this section, I demonstrate the performance of the model, in terms of accuracy (correct or not) and how this varies with the noise level and the decay and amplification parameters. I also show how the decay-amplify model integrates noise, and compare to a line attractor.

First, I determine how often the mechanism results in a correct response at the end of the delay period for different executive input times and different levels of noise. In all of these numerical experiments, the stimulus variable is 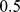, the delay period begins at time 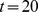 and ends at time 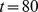. My criterion for success is that the activity at the end of the delay period (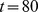) lies between 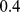 and 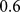 (so that 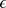 in equations (6) (8) is equal to 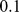).

**Figure 8:**
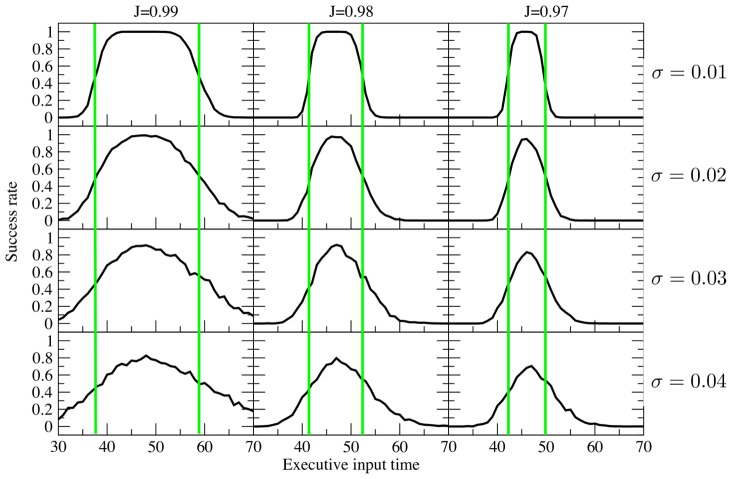
Model performance. Shown are the success rates for three decay rates (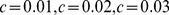) and four different noise strengths (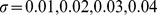) as a function of the timing of the executive input. For each decay, noise, and input value the simulation was run 200 times. The last 10 persistent cells in the feed forward chain were used in the calculation. The green lines show the boundaries of the timing interval described in the previous section. The noise causes a near symmetric distribution of end of delay period activity levels centered around the mean (the predicted value given in the previous section). At the boundaries, this distribution will be centered around the 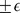 range required for success, and so the success rate at these boundaries is 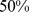 for all noise strengths.

In figure 8, I show the accuracy (as a percentage) of the model for three decay-amplify sets - 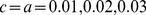. For a delay period of length 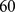, inequality (6) gives the bound on acceptable values for a line attractor:
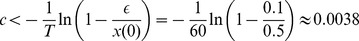
This corresponds to a coupling strength of (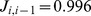). Expression (9) predicts an approximate interval width of 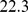 for 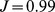, 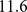 for 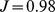, and 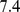 for 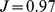. These are, respectively, 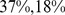, and 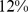 of the delay period lengths. Figure 8 clearly shows that the 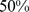 correctness rate lies at values that are consistent with this calculation. At the 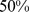 correctness rate, the mean (which is the trajectory discussed in the previous section) is right on the 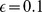 boundary. The noise causes a distribution of values for the activity level at the end point. If the network is tuned properly, the width of this distribution (variance) determines how reliable the recall will be. It is necessary to show that the decay-amplify model does not suffer from a noise integration disadvantage over a line attractor, otherwise the tuning advantageous would be nullified.

**Figure 9:**
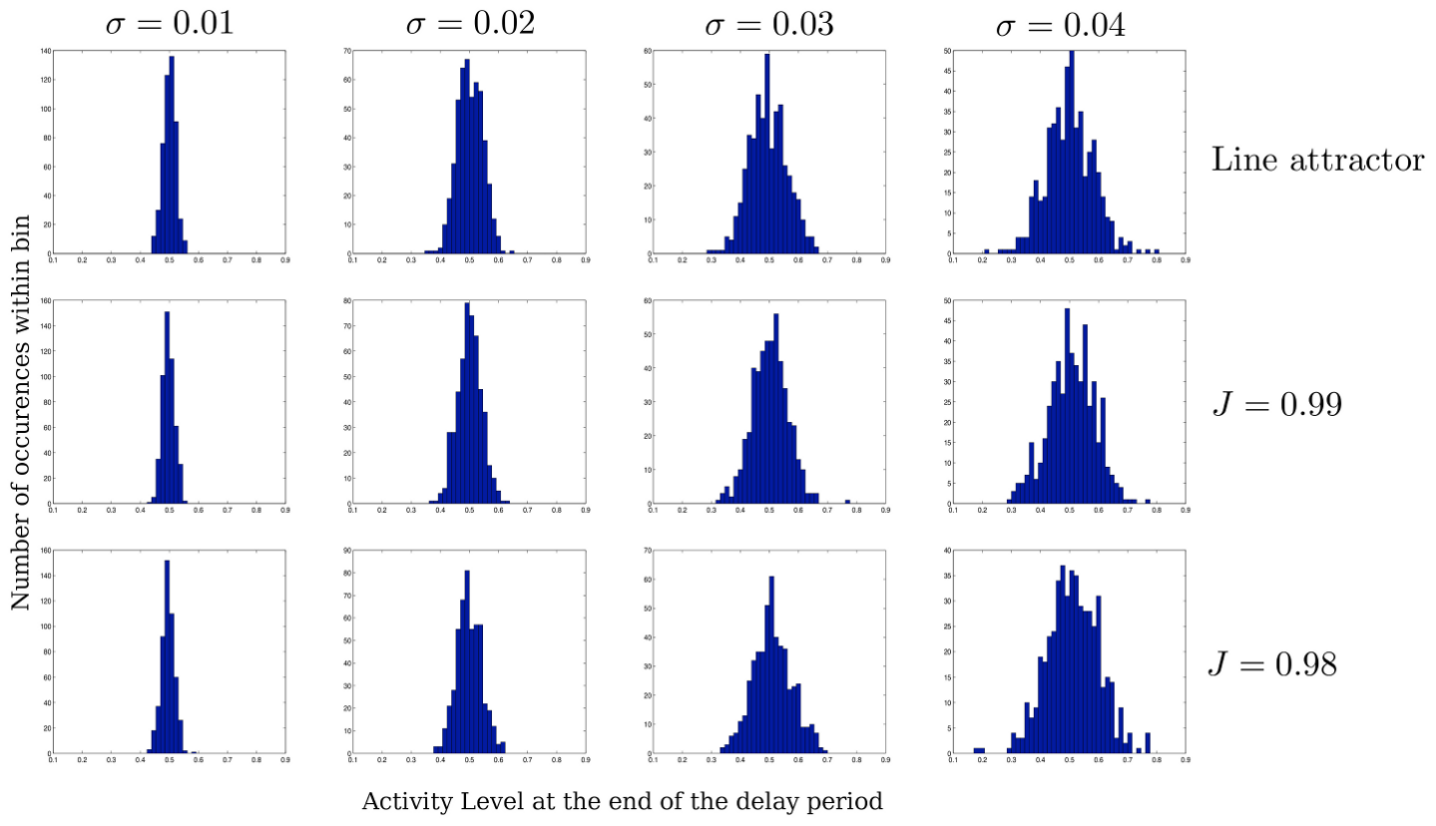
Distribution of end of delay period activity for a line attractor and two instances of the decay and amplify model. The first row shows the distributions for a line attractor. The second and third rows show the distributions for models with decay rates of 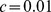 (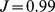), and 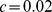 (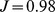), respectively. The corresponding amplification rates are also 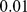 and 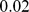. The distributions in a single column are very similar, demonstrating that the decay and amplify mechanism integrates noise no better or worse than a line attractor.

To determine the relationship between the cellular noise strength (the variance of the Wiener process) and the variance of the output, I ran the simulation 200 times, recording the activity at the end of the delay period for a single cell - the last persistent cell in the chain (cell #100).I repeat this for three instances of the model: A line attractor (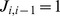) and two decay-amplify models with 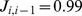 and 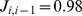. I simulate each of these with four different noise strengths (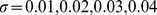). The results are shown in figure 9. The figure shows that the decay-amplify mechanism integrates noise no worse (or better) than a line attractor. This is not surprising. During the decay phase, the effect of noise is reduced. Conversely, during amplification the effect of noise is also amplified. The net effect of having a decay followed by an amplification of noise results in roughly the same distribution as a line attractor, where the effect of the noise uniform throughout the delay period.

### General model

I have demonstrated the decay and amplify mechanism using a simple feed forward model of neuronal activity. The choice of coupling was made to simplify the calculations and make the behavior of the model as transparent as possible. In this section, I describe a more general connectivity, where there is no bias, to demonstrate the decay-amplify mechanism is not dependent on a specific type of network architecture.

In place of the feed forward chain, I model the cells as
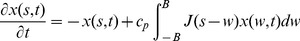
(10)where 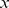 is the activity, 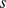 is location, 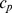 is the coupling strength, and 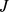 is a connectivity kernel. We assume that 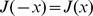 (symmetric), 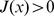, and 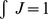 (on an unbounded domain, I use 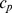 to scale for a bounded domain). For the simulations, I use
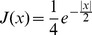
so that 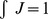. If 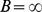 then if 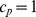 the networks admits a line attractor. Because we are considering a bounded domain, the value
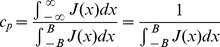
corresponds to the maintenance configuration in figure 1, where cells on the interior behave as a line attractor until the arrival of the wavefront. For simulations, the integral is discretized and 300 early/persistent cells are used.

**Figure 10:**
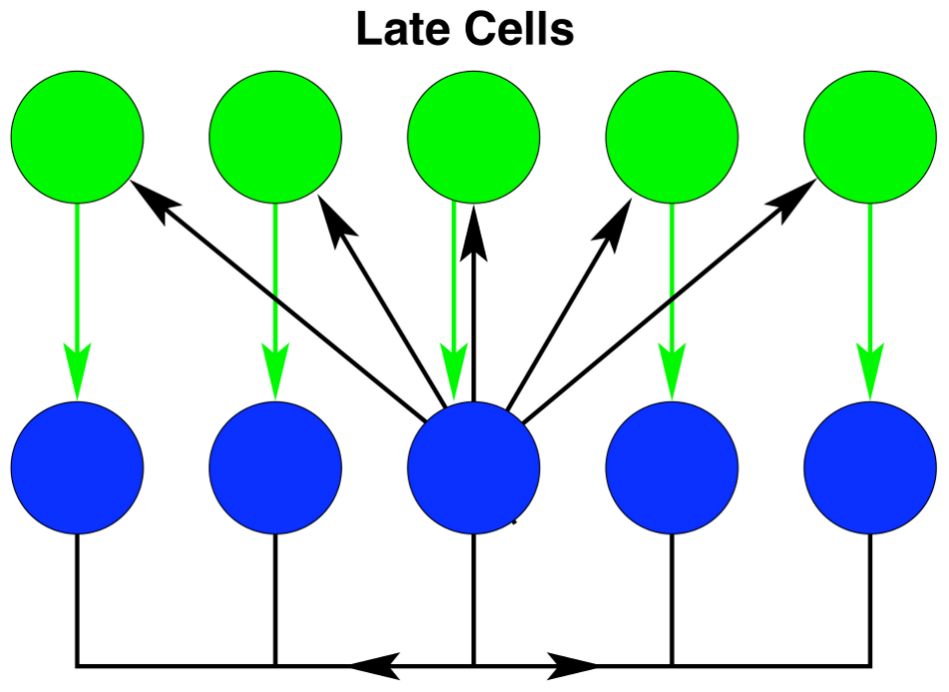
Schematic for the network described by equations (11–12). The blue circles represent the early and persistent cells through which the traveling wave propagates. The green circles represent the late cells. When the executive input arrives, the late cells are activated by the persistent cells. In this instance, the connections from the persistent to the late are divergent (black arrows). The late cells feed back onto the early and persistent cells in a one-to-one fashion (green arrows). This is only one of many possible implementations of the decay and amplify mechanism.

There are countless ways to implement the late cells. I choose to implement the late cells as a another line of cells, coupled to the activity described by (10) according to
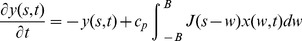
(11)and implement the feedback as one to one by rewriting equation (10) as
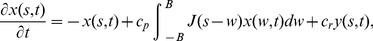
(12)where 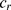 is the strength of the feedback from the late cells. Here, I have implemented the feedback from the late cells as a one to one relationship, and the connections from the early and persistent cells are divergent. Figure 10 shows the network schematic of this configuration.

**Figure 11:**
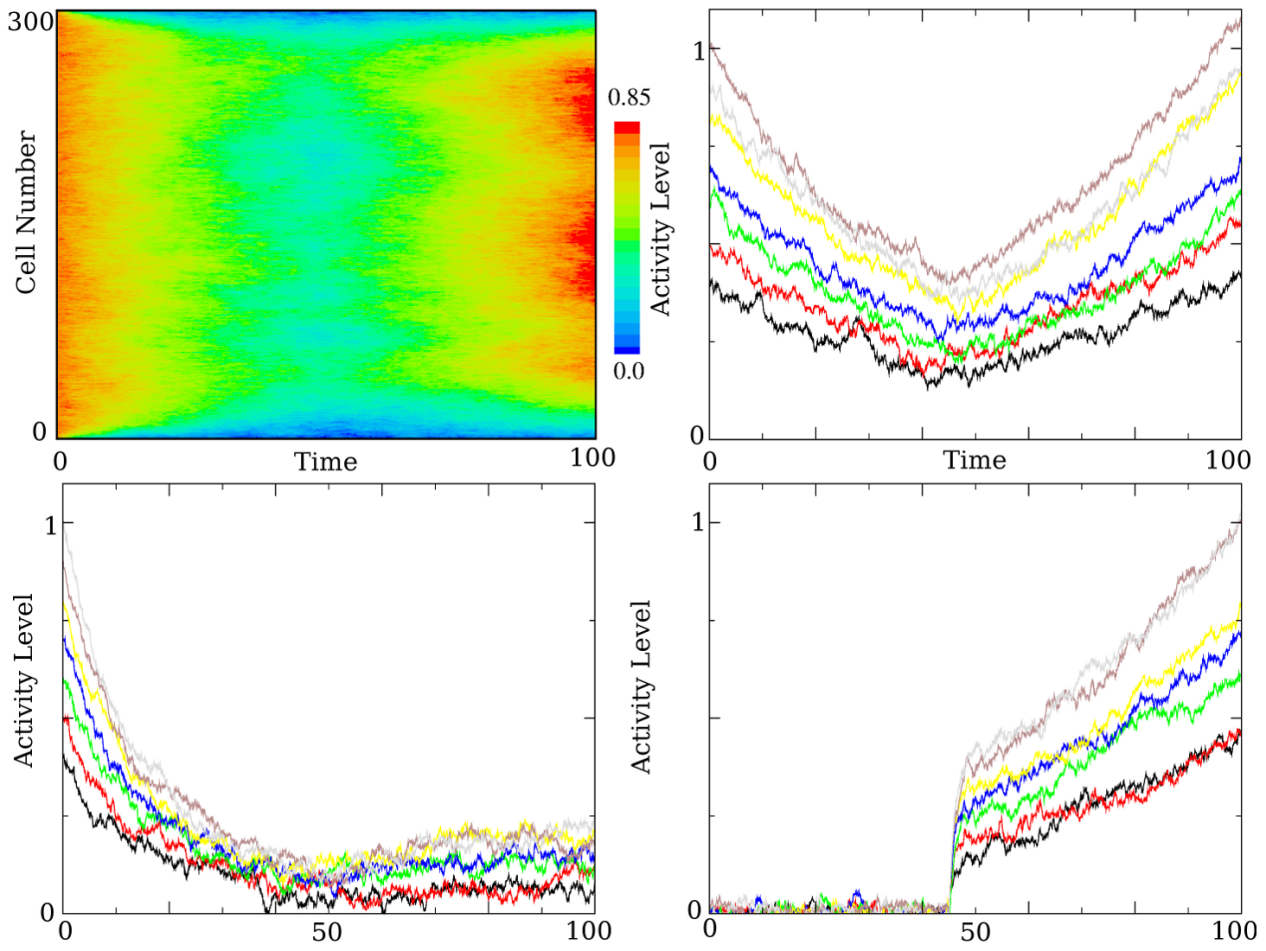
Simulations of the non-feed-forward model. The upper left panel is an array plot showing all early and persistent cells. Of note is the trailing wavefront that originates at the boundaries. Those cells that this wave front overtakes are the early cells. The upper right, lower left, and lower right panels show the evolution of a persistent, early, and late cell respectively, for a range of stimulus values (the loading phase is not shown). For all of these figures, the connection strength 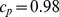, the feedback strength is 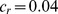, and the executive input arrives at 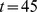.

Simulations of the network for a range of stimulus variable values are shown in figure 11. Shown are example time series for an early cell, a persistent cell, and a late cell, for a range of values of the stimulus variable. This figure demonstrates the decay-amplify model for a network that is not a feed-forward chain.

## Discussion

I demonstrate a mechanism that allows a network of cells to store an analog stimulus variable for a delay period, greatly easing the tuning requirements that would be necessary to accomplish the same feat with a line attractor. Using a simple mathematical representation of cellular activity, I demonstrate how wave fronts can account for the different types of activity observed in the experiments [1], [2] (early, persistent, and late), the systematic change in the number of cells encoding the stimulus, as well as the in-trial variability of persistent cells. The keystone of the mechanism is an external signal that is executive in nature and provides timing to the network.

I show that the proposed mechanism eases the requirements on cell-to-cell connections by initially allowing the cellular activity to decrease. A subsequent amplification, initiated by the executive input, corrects for the decay. I show that the restriction on the timing of the executive input depends on the decay and amplification rates in a way that is feasible for networks in the brain, even when the decay and amplification rates are well outside those allowable for a line attractor. The memory mechanism that I describe has an additional advantage: It allows the network to quickly adapt to delay periods of different lengths.

The tuning strategy involves changing the time when a wave front is allowed to propagate into a previously response-less group of cells, late cells, which do not encode the stimulus until the latter half of the delay period. This strategy agrees with the data shown in [1], where upon lengthening the delay period from 3 seconds to 6 seconds, the activation of late cells is pushed back. Importantly, this transition is not accomplished in one step, but rather over a series of steps. Consistent with this gradual transition is an increase in the error rate for a few trials after the change of delay period length takes place [1]. The mechanism that I describe suggests that there should be an increase in the error rate upon changing the length from 6 to 3 seconds - a testable hypothesis that is somewhat counter-intuitive.

The analysis that I provide is for a feed forward network. This choice for the connectivity matrix 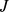 was made so that the behavior of the model is as transparent as possible. I make the claim that this choice of connectivity is not crucial for the mechanism to work, and I demonstrate the mechanism using a recurrent network. In general, a linear filter that slowly decays the signal (the eigenvalues of the network are negative, with a few near 0 for slowness -ie. near a line attractor) will work for the mechanism, since all that is needed is that monotonicity be preserved. The evolution of early and persistent cells depends on the relative decay rates. If the network of linear filters has a range of decay rates which are spatially localized, then the activity level of some cells will decay quickly, while the activity level of others will decay slowly. Those that decay quickly are candidates to be early cells. Those that decay slowly maintain a monotonic relationship with the stimulus and are more likely to be persistent cells. Any such network that is not normal can be viewed as a feed forward network under an appropriate change of basis [9]. If the network is normal, then the behavior can be viewed as independent modes (the eigenvectors) and will not be, strictly speaking, feed forward. Such a network still is capable of various decay rates, and so can admit early and persistent cells just as a feed forward network can.

I treat late cells as a distinct group, defined by the inability to respond to input until an executive input is received. There are many ways to implement the late cells. In the feed forward model, I attach them to the end of the chain. In the symmetric model, I treat them as a separate line of cells that are reciprocally connected with the early and persistent cells. In either case, once allowed to participate in the task, they assume the activity of the persistent cells, and amplify the network. Any late cell configuration that does this will work, there are no other requirements on the late cells.

A major claim of this paper is that the late cells are governed by a timing input, executive in nature. Prior to the arrival of the executive signal, these cells do not respond to input. There are many plausible mechanisms for this. The most obvious to me is a shunt, as described by Torre and Poggio [15]. If ion channels that have a reversal potential near the membrane resting potential and are held open, the impact of other channel openings (eg. sodium) will be greatly reduced. So, in this scenario, the action of the executive input would be to remove this shunt by allowing the responsible channels to close. Another possibility is inhibition. Inhibitory control is known to be an important element of prefrontal function [12]. A constant inhibitory drive onto the late cells would hold them quiescent. Removal of this inhibition would serve the purpose of the executive input.

There have been numerous modeling studies of the delayed discrimination experiments. When comparing and contrasting the proposed decay-amplify mechanism with these models, I focus on the three most important features: 1). The decay-amplify model is an extension of a line attractor. 2). The proposed model accounts for the division into early, persistent and late cells using a wave front.3). An external executive input is used as a timing mechanism for the model. This external input is independent of the stimulus variable.

A line attractor is a natural choice to store the value of an analog variable. Other authors have used a line attractor to model the Romo data [7], [16]. In [7], Machens et al. use the interplay between cells that respond to the stimulus in different ways (monotonically increasing relationship versus a decreasing relationship with the stimulus variable) and inhibition to form the attractor. Singh and Eliasmith [17] use a “neural integrator”, which is similar to a line attractor in that the output of the system does not change without a change in the input, and that the connection strengths between cells needs to be precise. The decay-amplify model is a novel extension of these models. Rather than require the very tight tuning necessary for a line attractor, I allow the cellular activity level to drift slowly. The value of the stimulus variable is lost, but the activity of the decaying system maintains the monotonic relationship with the stimulus variable.

It is important to note that the line attractor has not been the only proposed means of modeling the data. Barak et al. [16] explore two types of models, in addition to a line attractor. They show that a network with random connectivity can perform the task by using a linear sum over the constituent neurons. They also demonstrate a learning model that begins like the random network (random connections, linear readout) but then adjusts the connection strengths between neurons based on past performance. Each of these models are capable of the performing the task, though none of them account for the late cells. Additionally, a change in the length of the delay period would require a complete recalculation of the weights applied in the linear readout scheme, rather than changing a single parameter.

There have been studies that use completely different strategies to store the stimulus variable. Miller et al. [18] tune a model so that it approximates a line attractor near a degeneracy. The attractor holds the memory by holding the activity level constant for the duration of the delay period. Miller includes inhibition and cells that encode the stimulus variable both positively and negatively. The attractor is formed through the interplay of these different cells. Another strategy that has been described is to store the stimulus as a level of facilitation in the cells [19], [20]. The initial stimulus facilitates the synaptic connections between cells, and these facilitated cells later respond to a recall signal. The facilitation decays slowly, so that the memory is stored at the synaptic level. Neither of these models attempt to describe the diversity of the cellular responses, in particular the division into early, persistent and late cells.

Barak et al. [20] show that the stimulus information is held in a dynamic way. They quantify a population state for the recorded cells by trends in how the cells are tuned to the stimulus. They look at two representations of the population state, sensory and memory. The sensory representation of the stimulus is the population state at the beginning of the delay period. The memory representation is the population state at the end of the delay period. The authors show that applying the sensory representation to cells at the end of the delay period, or applying the memory representation to the cells at the beginning of the delay period, provides no stimulus information. Thus, they demonstrate that the stimulus information is held in a dynamic way, by different cells at different times. Those neurons that are classified as sensory correspond to the early cells in the model I propose. At the beginning of the delay period, they are tightly tuned with the stimulus. At the end of the delay period, they are devoid of information. Similarly, the late cells begin with no stimulus information, but gain it later in the delay period. The authors note that the classification of sensory or memory is only weakly correlated with the classification of early or late. The model that I propose is too simple to account for all of the data, but the wave front hypothesis for diversity of responses neatly accounts for the transition from a sensory representation to a memory one, as described.

Singh and Eliasmith [17] offer an alternative mechanism to account for the diversity. They build a network of cells, each having a preferred orientation to a state space variable. As the state space variable evolves, it passes through the tuning curves of the cells. The distribution of preferred orientations yields a diverse array of responses including early, persistent (ramping type, as described in [4]), and late.

The centerpiece of the decay-amplify mechanism is an executive input that adds timing to the network. This is a novel addition to the modeling literature, though the separation of stimulus and time has been explored before. Use of an external executive input for timing purposes is in agreement with other studies that separate the stimulus component of the activity from a time component. Machens [14] shows that there are two separate causes of variance in the data: stimulus and time. They show that the variance attributed to time is likely external in origin. This strongly supports the use of an external executive input to time the network.

Singh and Eliasmith [17] also separate stimulus and time. The state space variable that evolves through the tuning curves has two components, the stimulus and a variable that is akin to elapsed time. They do not implement their timing component as an external signal. Moreover, they model it as a passive process that is ongoing throughout the delay period. In contrast, the executive input that I propose is external, and upon arrival the behavior of the network drastically changes. These drastic network changes can be seen in the data, where there is an obvious change in behavior near the mid point of the delay period [1]. The number of tuned cells begins to increase, and the behavior of individual cells changes. The tuning curves in the Singh and Eliasmith model are monotonic along both axis (stimulus and time), and so the activity pattern that is the focus of this paper, a decay of activity followed by an amplification, is not possible in the Singh-Eliasmith model without an external intervention.

**Figure 12:**
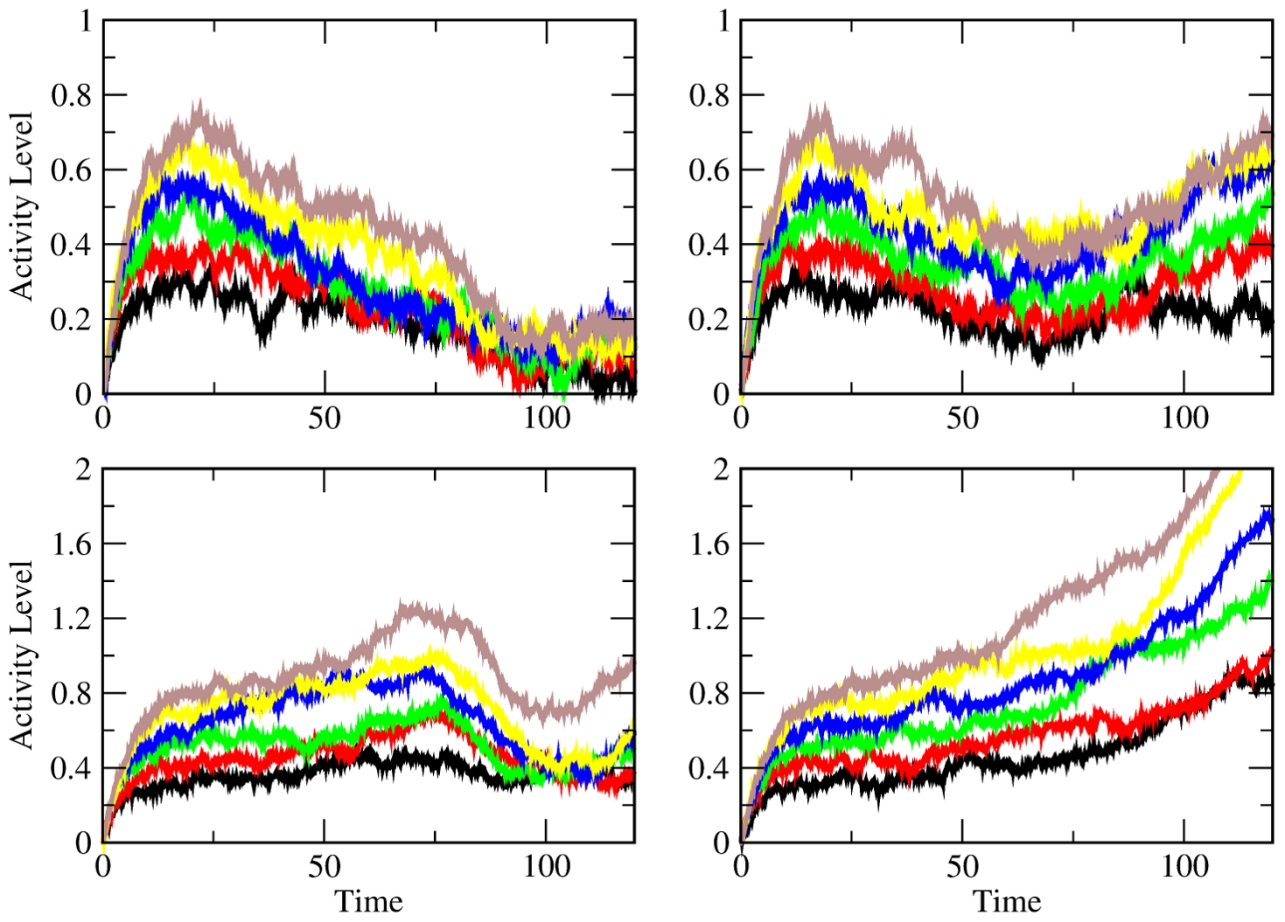
The four different activity patterns observed in the experiments generated using the model (1). In all panels, the stimulus was removed at 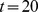. In the upper panels, the entries of 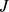 are 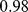, so that there is a decline in activity following the removal of the stimulus. In the left panel, the feedback is weak so the decrease continues after the executive input has arrived. In the right, the feedback is strong enough to increase the activity of the persistent neuron. The lower panels have coupling strengths of 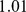, so that there is an initial increase in activity after the stimulus is removed. The lower left panel shows a cell that gets caught by the trailing wave front. The right panel shows a cell that continues to rise throughout the delay period because it is not caught by the front prior to the end of the delay period.

The decay-amplify model is the only model that directly addresses the two-mode behavior that Brody et al. [1] describe - behavior that is different during the first half of the delay period than during the second half. I focus on activity that decreases during the beginning of the delay period and then is amplified to recover the stimulus information. Another type of persistent activity that occurs is ramping, where a cell either increases or decrease for the duration of the delay period while maintaining a monotonic relationship with the stimulus variable [3], [4]. The simple linear filter that I use is capable of generating all of these types of behavior (figure 12), but they cannot coexist for the simple chain of neurons that I describe.

In conclusion, this study suggests another potential means of storing a stimulus variable as a firing rate for the duration of a delay period. This mechanism stands apart from previous models that do not take the variability during the delay period into account. Moreover, this variability is revealed as part of the solution to the memory problem, rather than a confound. The major claim that I make is that there is an external timing signal that causes the network to switch modes. There are many features of the data that I do not take into account (eg. inhibition and different monotonic encodings). These features are almost certain to play a role in the prefrontal calculations, and figuring out how everything works together is an ongoing process.

## Supporting Information

Code S1 Implementation of the feed forward network, as described by (3–4).) I have included the file mem_net.txt. This is a file for use with the dynamical systems software XPPAUT [21].
(TXT)
